# Supplementary material for: Casein kinase 1a mediates a two-step subunit remodeling mechanism to regulate the FRQ-FRH circadian clock complex
Source: Nat Commun. 2026 Jan 8;17:418. doi: 10.1038/s41467-025-68087-4 (PMC12796302; doi:10.1038/s41467-025-68087-4)
Supplement: Supplementary file 1 — Supplementary Information [file 41467_2025_68087_MOESM1_ESM.pptx]

## Slide 1
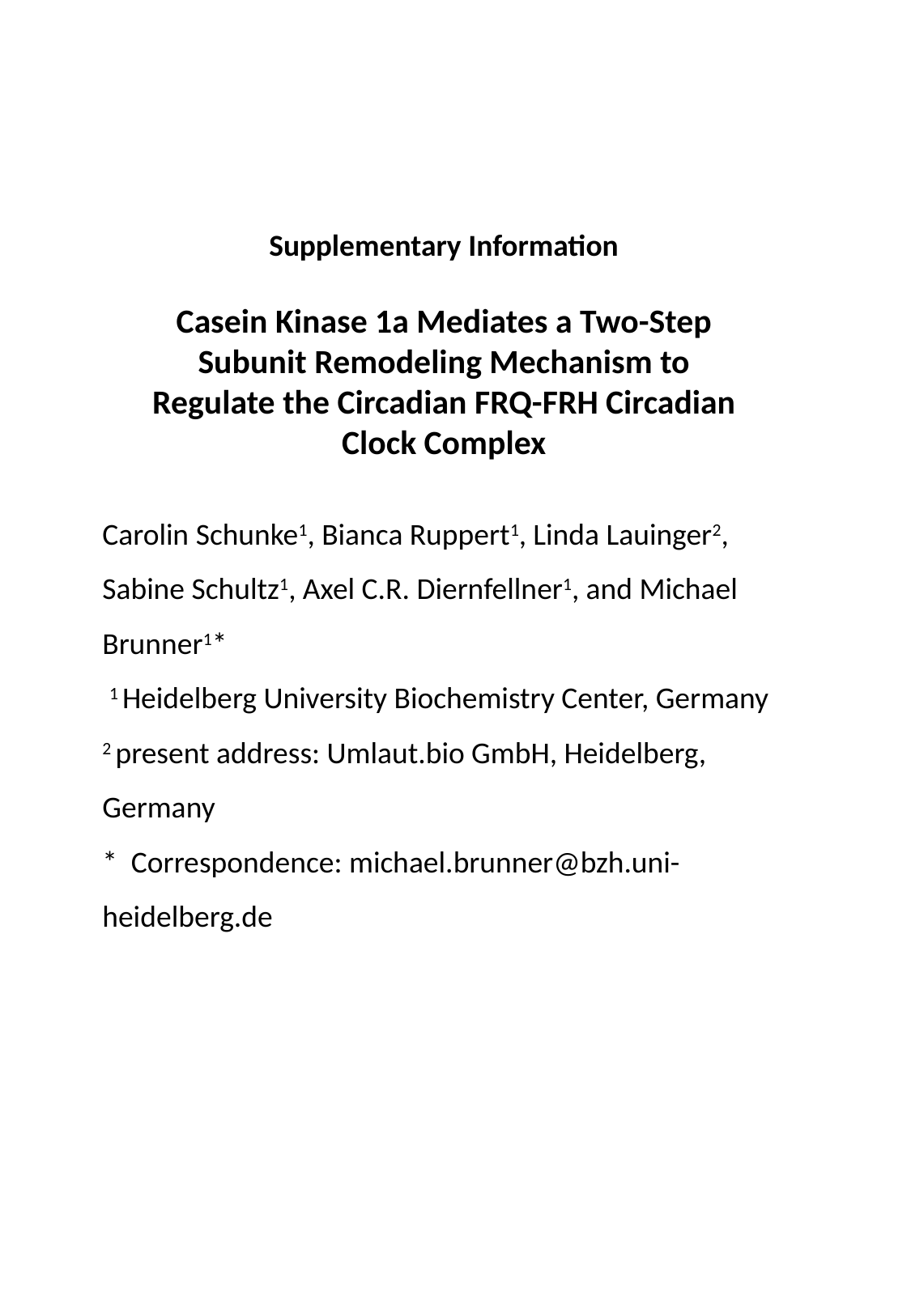

Supplementary Information
Casein Kinase 1a Mediates a Two-Step Subunit Remodeling Mechanism to Regulate the Circadian FRQ-FRH Circadian Clock Complex
Carolin Schunke1, Bianca Ruppert1, Linda Lauinger2, Sabine Schultz1, Axel C.R. Diernfellner1, and Michael Brunner1*
 1 Heidelberg University Biochemistry Center, Germany
2 present address: Umlaut.bio GmbH, Heidelberg, Germany
* Correspondence: michael.brunner@bzh.uni-heidelberg.de

## Slide 2
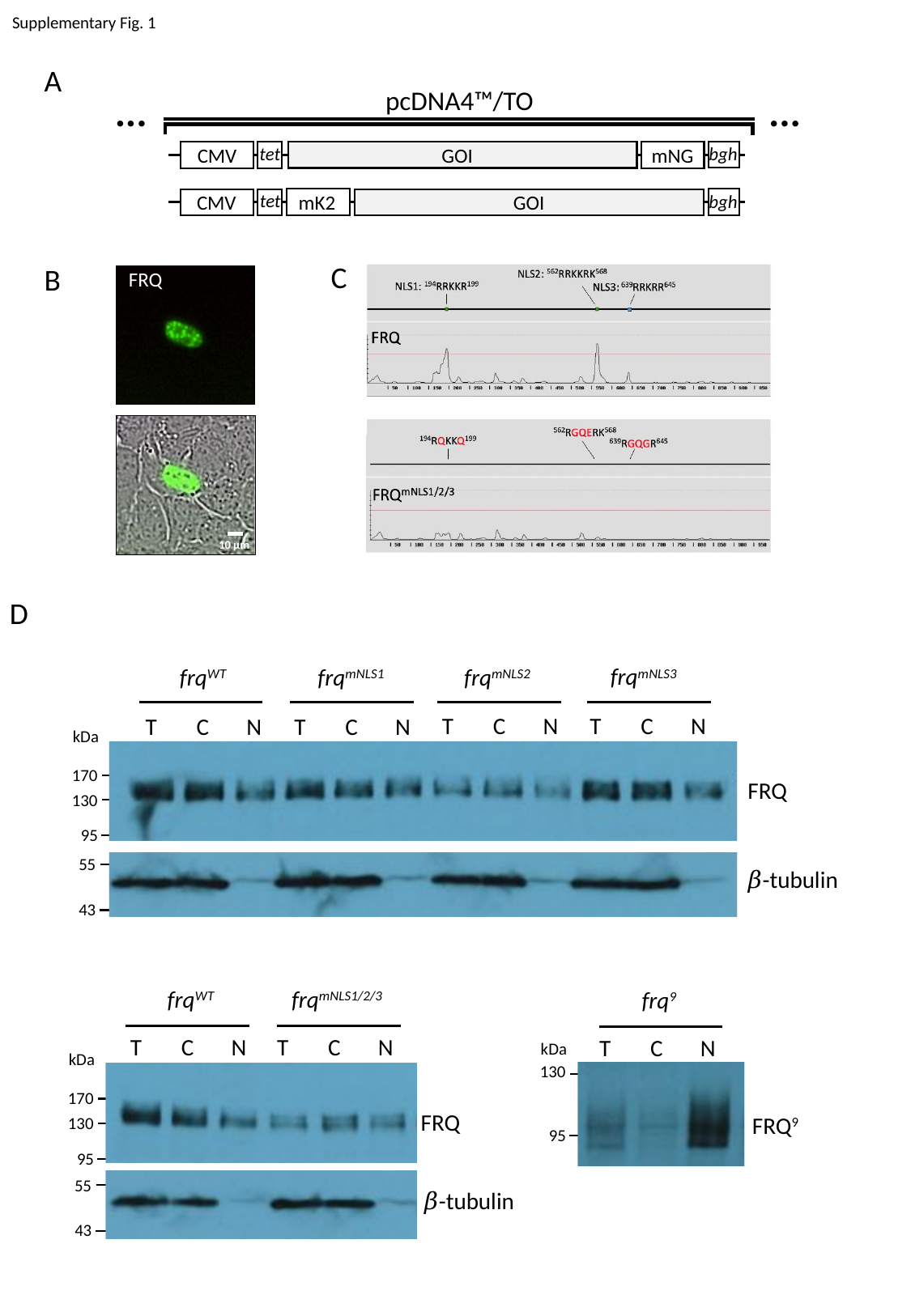

Supplementary Fig. 1
A
pcDNA4™/TO
...
...
CMV
tet
GOI
bgh
mNG
tet
bgh
CMV
mK2
GOI
C
B
FRQ
10 µm
10 µm
D
frqmNLS3
frqmNLS2
frqmNLS1
frqWT
T
C
N
T
C
N
T
C
N
T
C
N
kDa
170
FRQ
130
95
55
𝛽-tubulin
43
frqWT
frqmNLS1/2/3
T
C
N
T
C
N
kDa
170
FRQ
130
95
55
𝛽-tubulin
43
frq9
T
C
N
kDa
130
FRQ9
95

## Slide 3
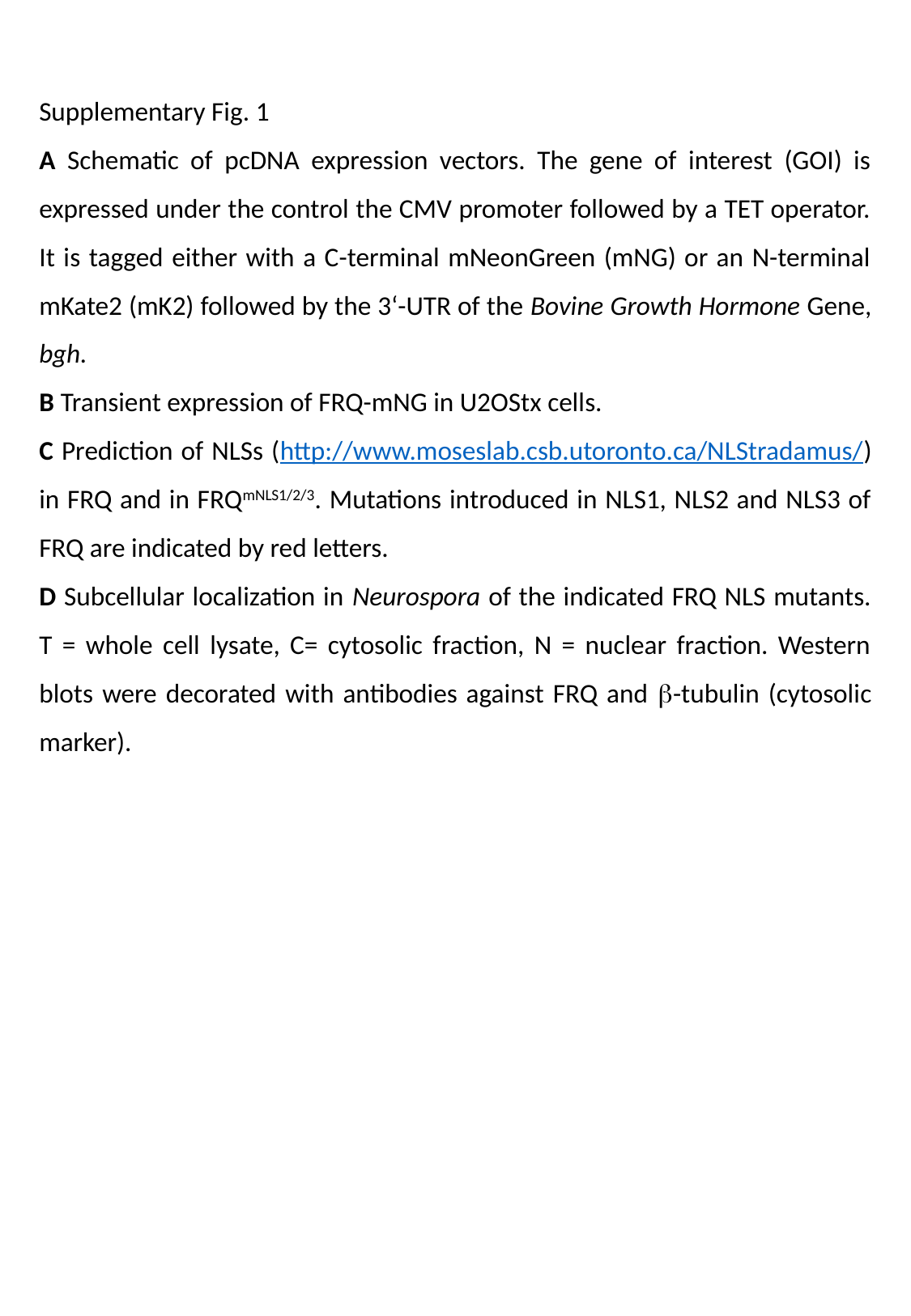

Supplementary Fig. 1
A Schematic of pcDNA expression vectors. The gene of interest (GOI) is expressed under the control the CMV promoter followed by a TET operator. It is tagged either with a C-terminal mNeonGreen (mNG) or an N-terminal mKate2 (mK2) followed by the 3‘-UTR of the Bovine Growth Hormone Gene, bgh.
B Transient expression of FRQ-mNG in U2OStx cells.
C Prediction of NLSs (http://www.moseslab.csb.utoronto.ca/NLStradamus/) in FRQ and in FRQmNLS1/2/3. Mutations introduced in NLS1, NLS2 and NLS3 of FRQ are indicated by red letters.
D Subcellular localization in Neurospora of the indicated FRQ NLS mutants. T = whole cell lysate, C= cytosolic fraction, N = nuclear fraction. Western blots were decorated with antibodies against FRQ and β-tubulin (cytosolic marker).

## Slide 4
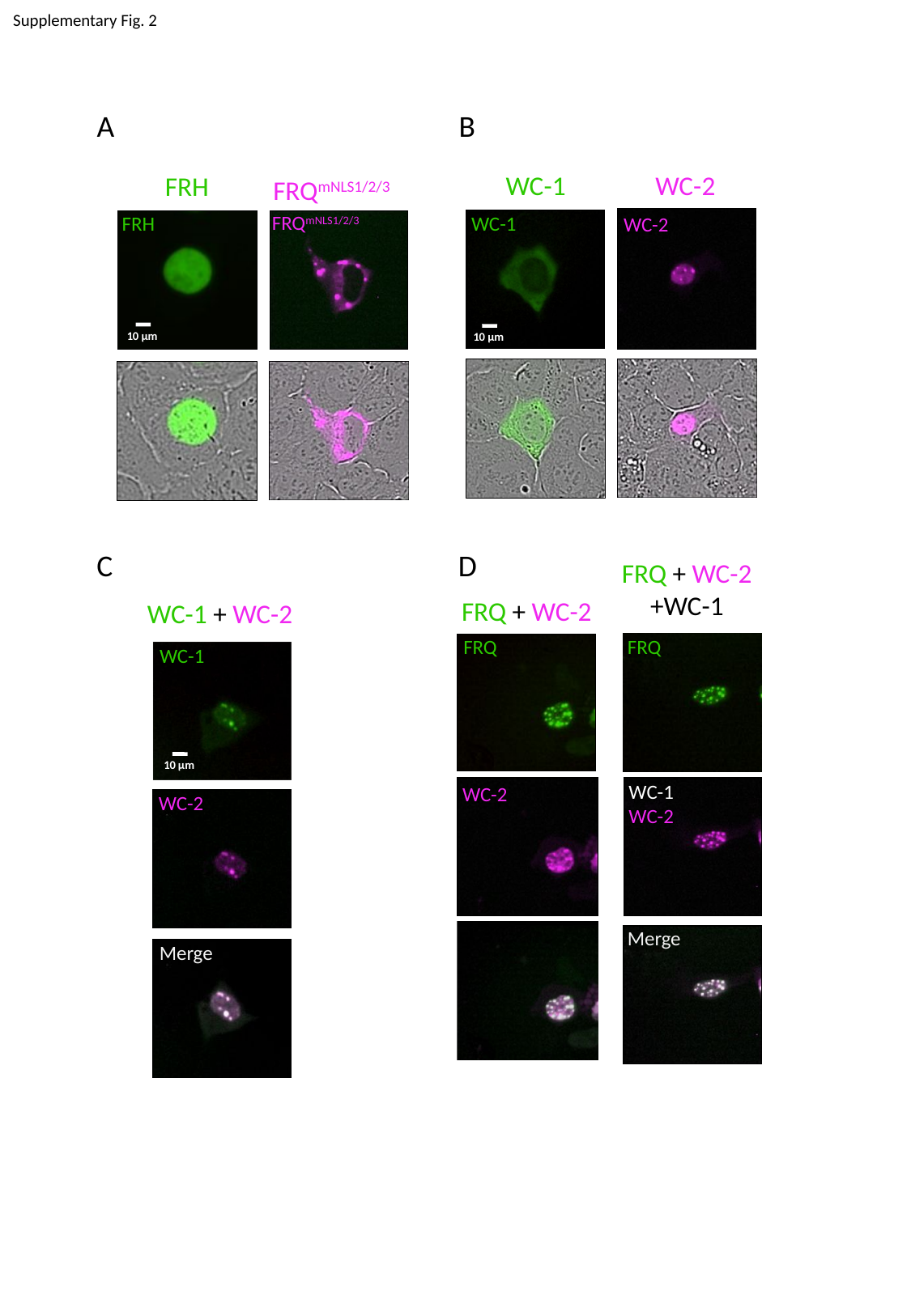

Supplementary Fig. 2
A
B
WC-1
WC-2
WC-1
WC-2
10 µm
10 µm
FRH
FRQmNLS1/2/3
FRQmNLS1/2/3
FRH
10 µm
C
D
FRQ + WC-2
+WC-1
FRQ + WC-2
FRQ
FRQ
WC-1
WC-2
Merge
WC-2
WC-1 + WC-2
WC-1
10 µm
WC-2
Merge
10 µm

## Slide 5
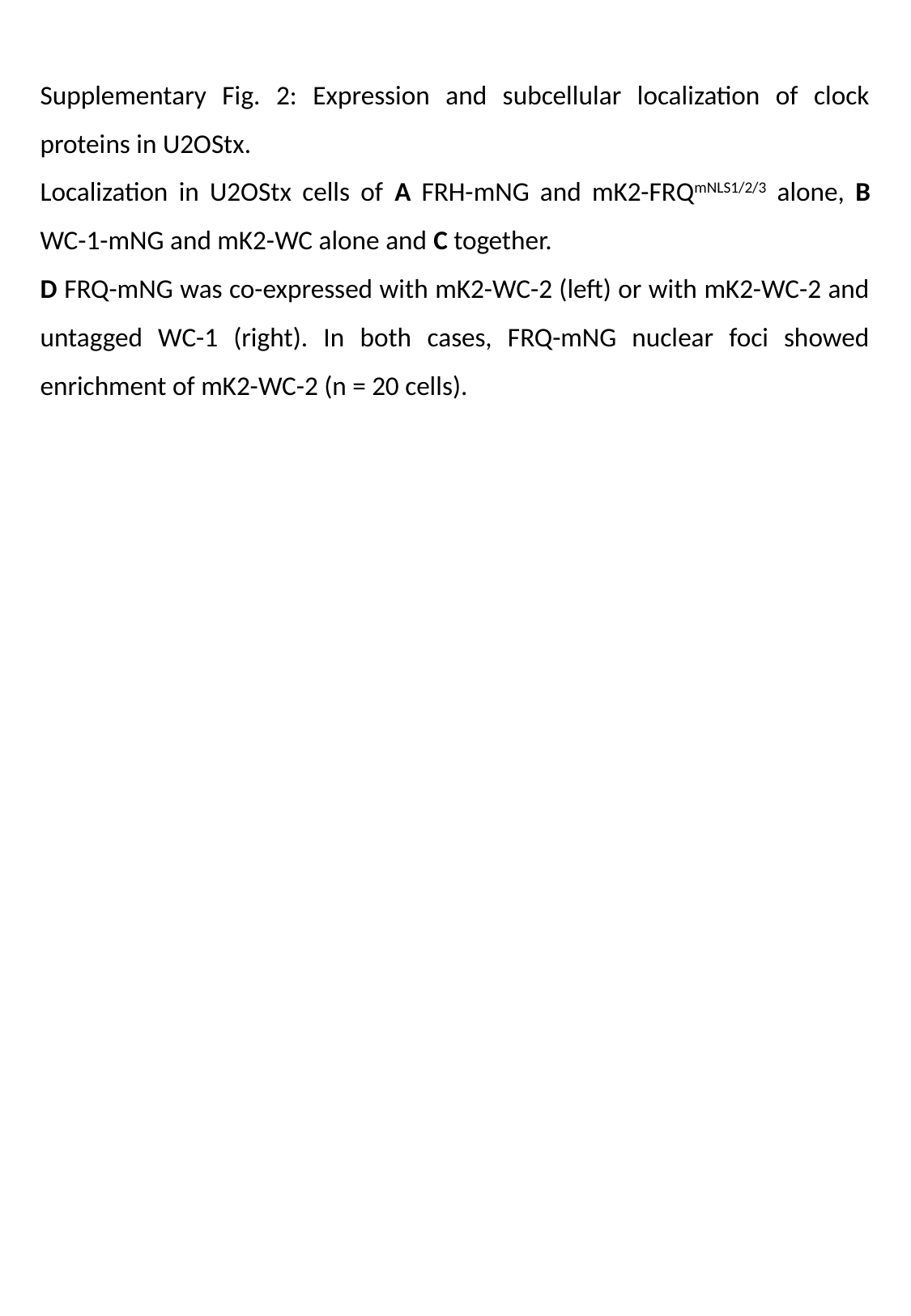

Supplementary Fig. 2: Expression and subcellular localization of clock proteins in U2OStx.
Localization in U2OStx cells of A FRH-mNG and mK2-FRQmNLS1/2/3 alone, B WC-1-mNG and mK2-WC alone and C together.
D FRQ-mNG was co-expressed with mK2-WC-2 (left) or with mK2-WC-2 and untagged WC-1 (right). In both cases, FRQ-mNG nuclear foci showed enrichment of mK2-WC-2 (n = 20 cells).

## Slide 6
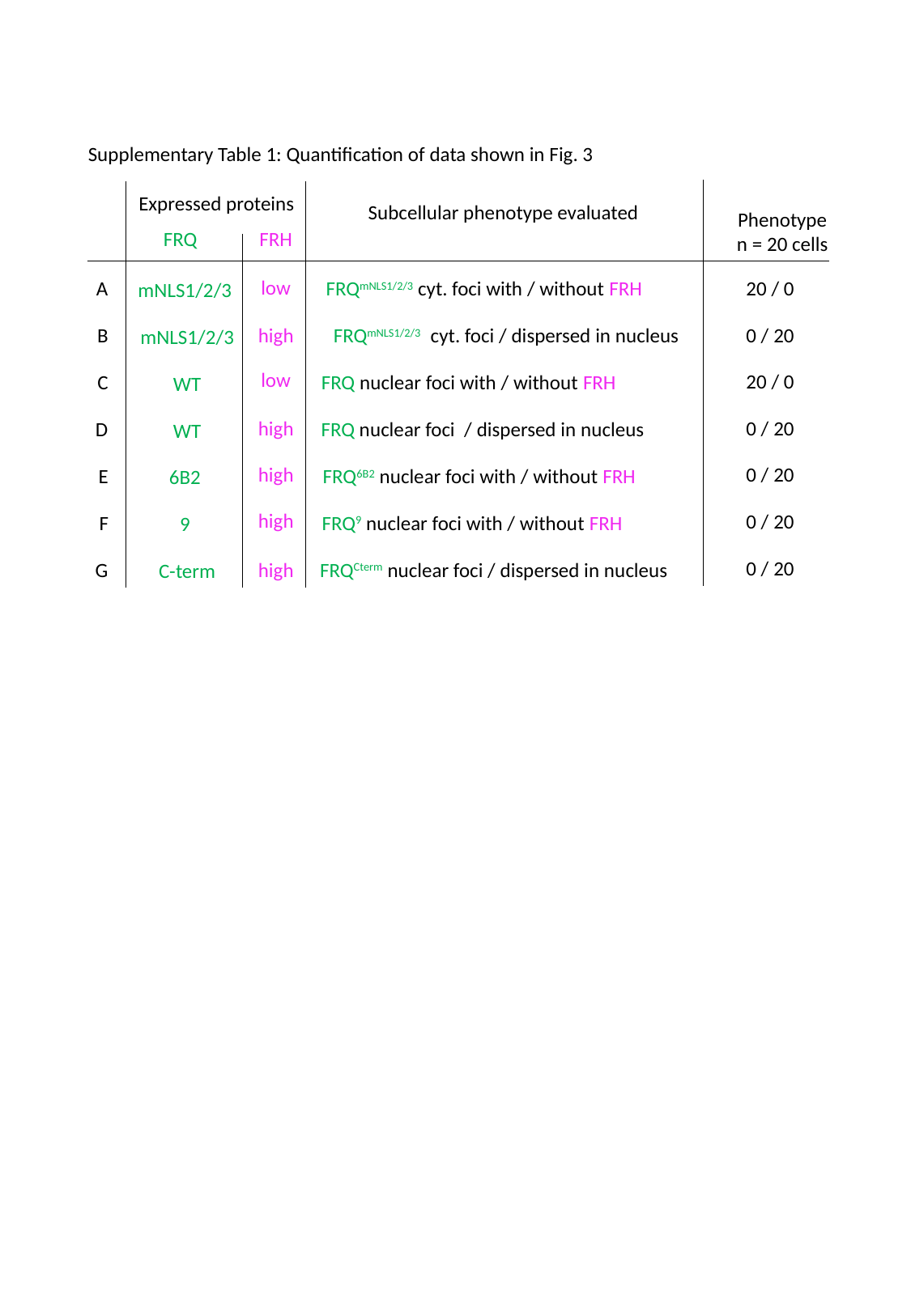

Supplementary Table 1: Quantification of data shown in Fig. 3
Expressed proteins
Subcellular phenotype evaluated
Phenotype
n = 20 cells
FRQ
FRH
low
mNLS1/2/3
high
mNLS1/2/3
low
WT
high
WT
high
6B2
high
9
high
C-term
FRQmNLS1/2/3 cyt. foci with / without FRH
FRQmNLS1/2/3 cyt. foci / dispersed in nucleus
FRQ nuclear foci with / without FRH
FRQ nuclear foci / dispersed in nucleus
FRQ6B2 nuclear foci with / without FRH
FRQ9 nuclear foci with / without FRH
FRQCterm nuclear foci / dispersed in nucleus
A
B
C
D
E
F
G
20 / 0
0 / 20
20 / 0
0 / 20
0 / 20
0 / 20
0 / 20

## Slide 7
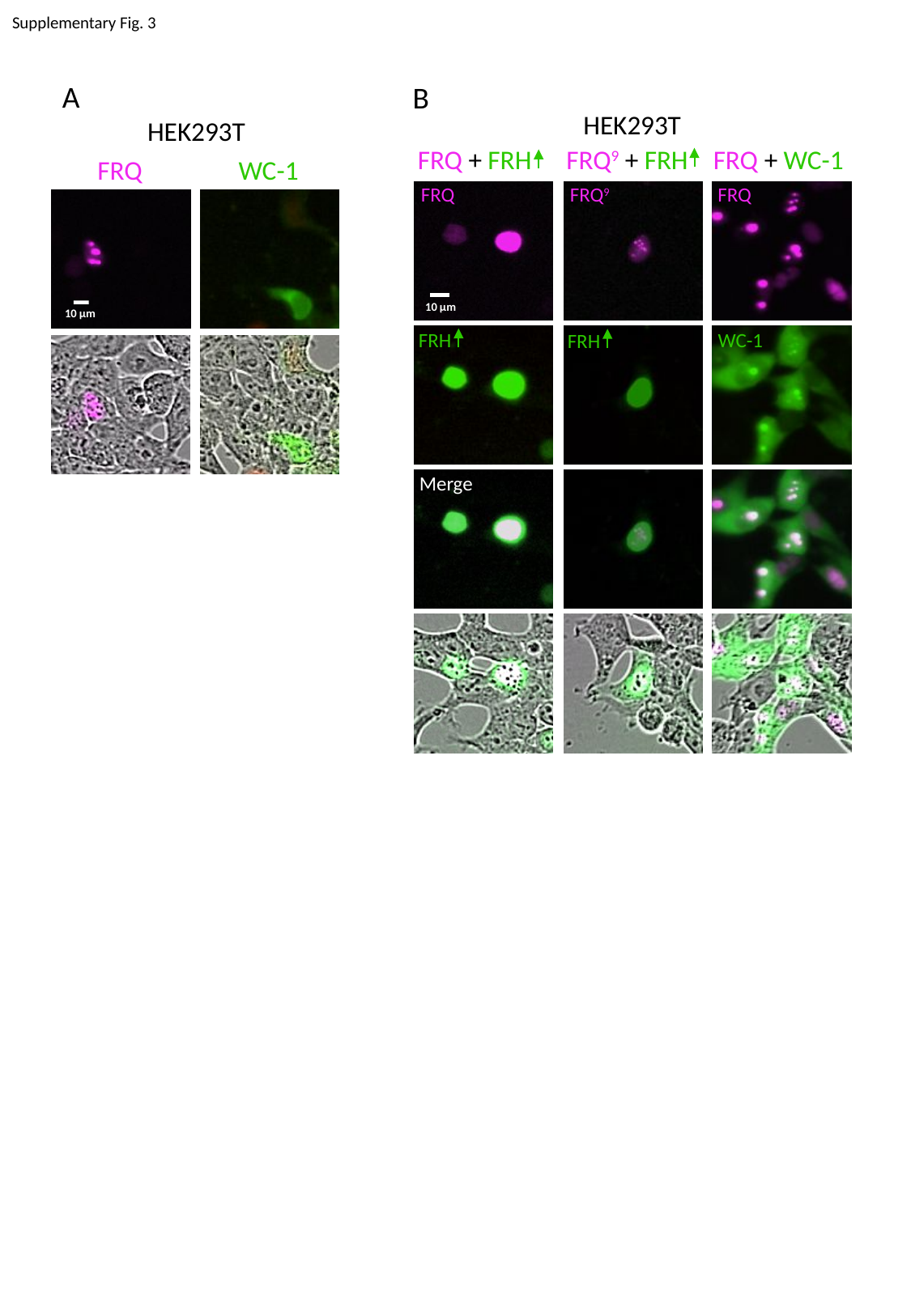

Supplementary Fig. 3
A
B
HEK293T
FRQ + FRH
FRQ9 + FRH
FRQ + WC-1
FRQ
FRQ9
FRQ
10 µm
WC-1
FRH
FRH
Merge
HEK293T
FRQ
WC-1
10 µm

## Slide 8
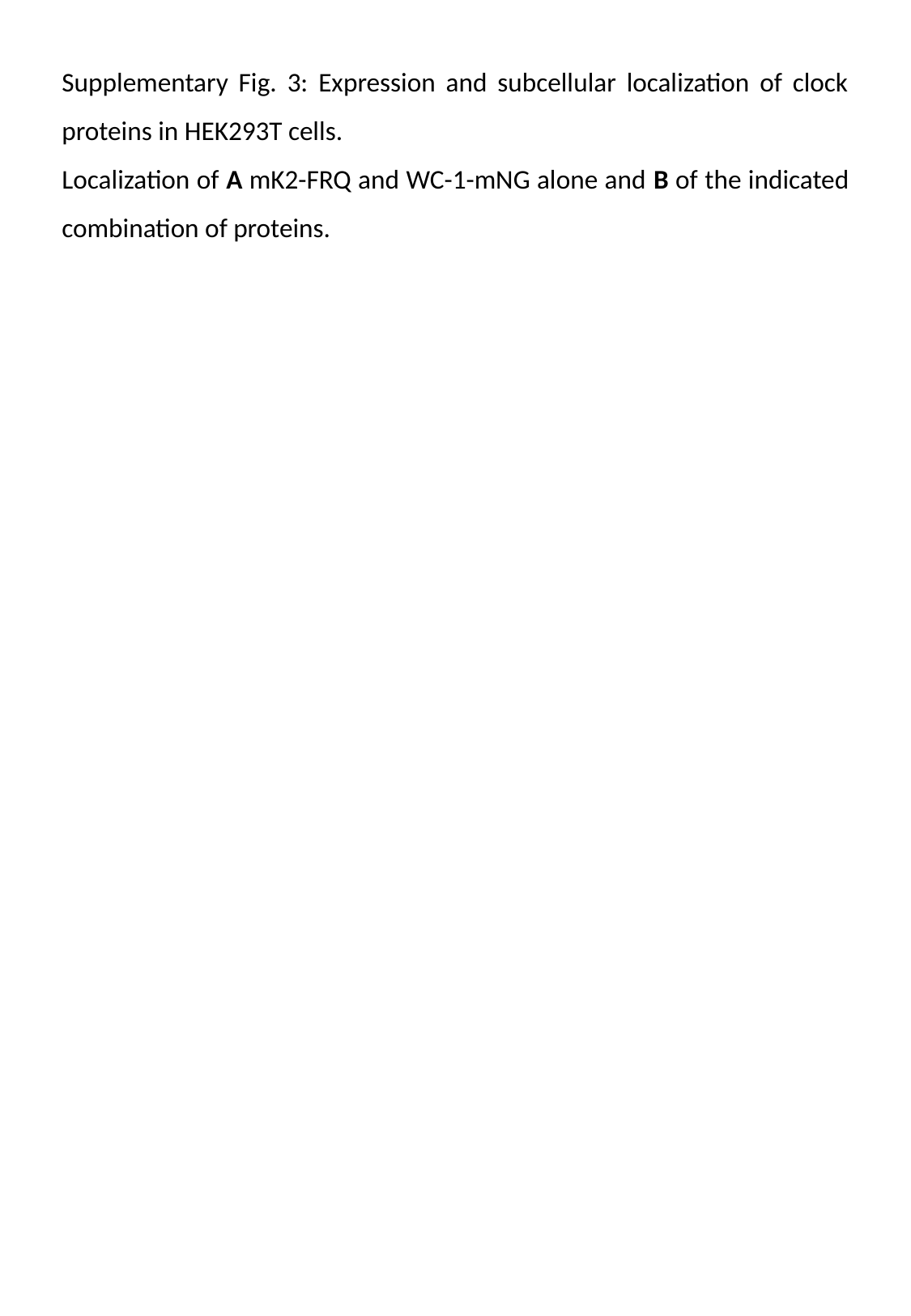

Supplementary Fig. 3: Expression and subcellular localization of clock proteins in HEK293T cells.
Localization of A mK2-FRQ and WC-1-mNG alone and B of the indicated combination of proteins.

## Slide 9
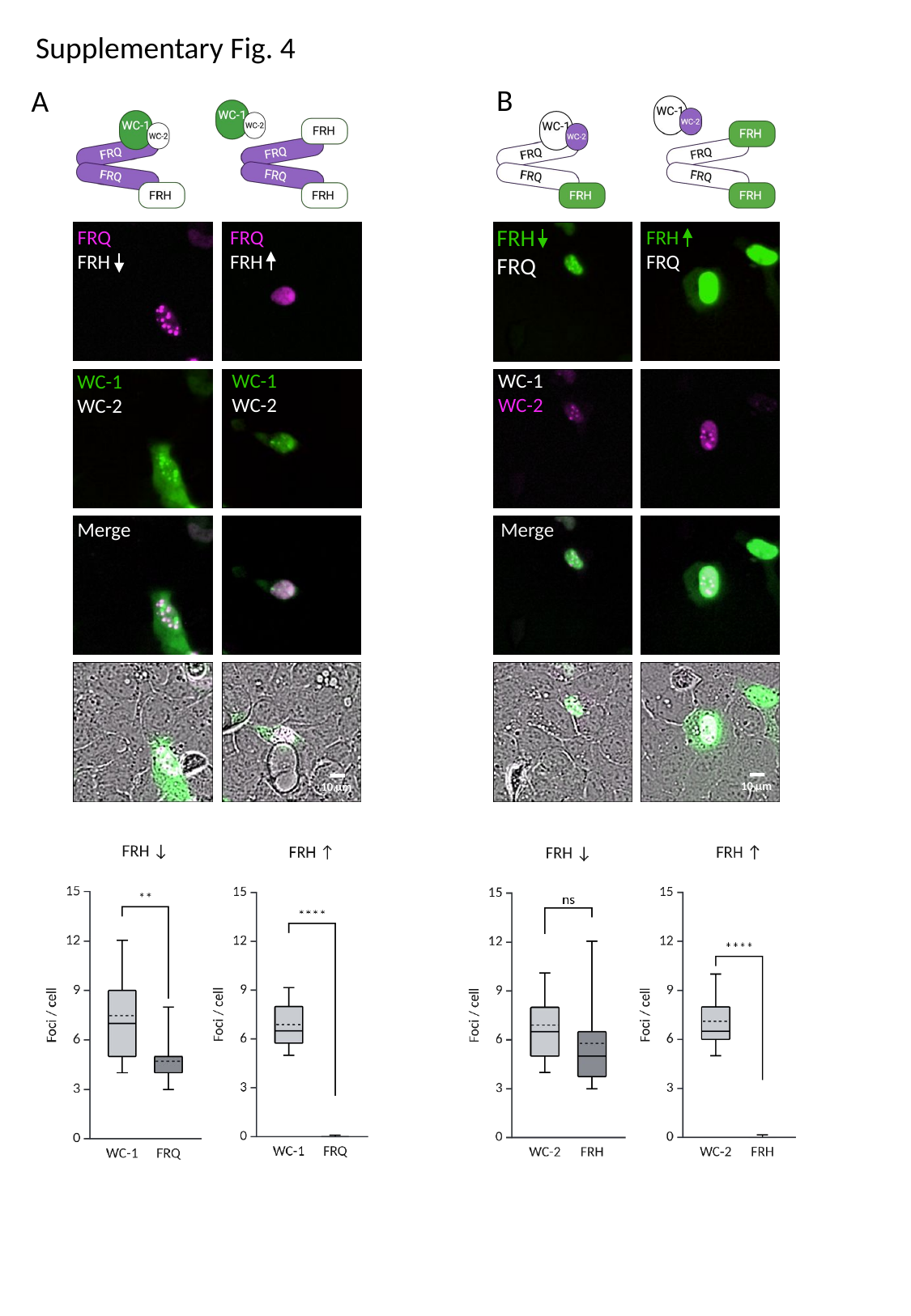

Supplementary Fig. 4
B
A
FRH
FRQ
FRH
FRQ
WC-1
WC-2
Merge
10 µm
FRQ
FRH
FRQ
FRH
WC-1
WC-2
WC-1
WC-2
Merge
10 µm

## Slide 10
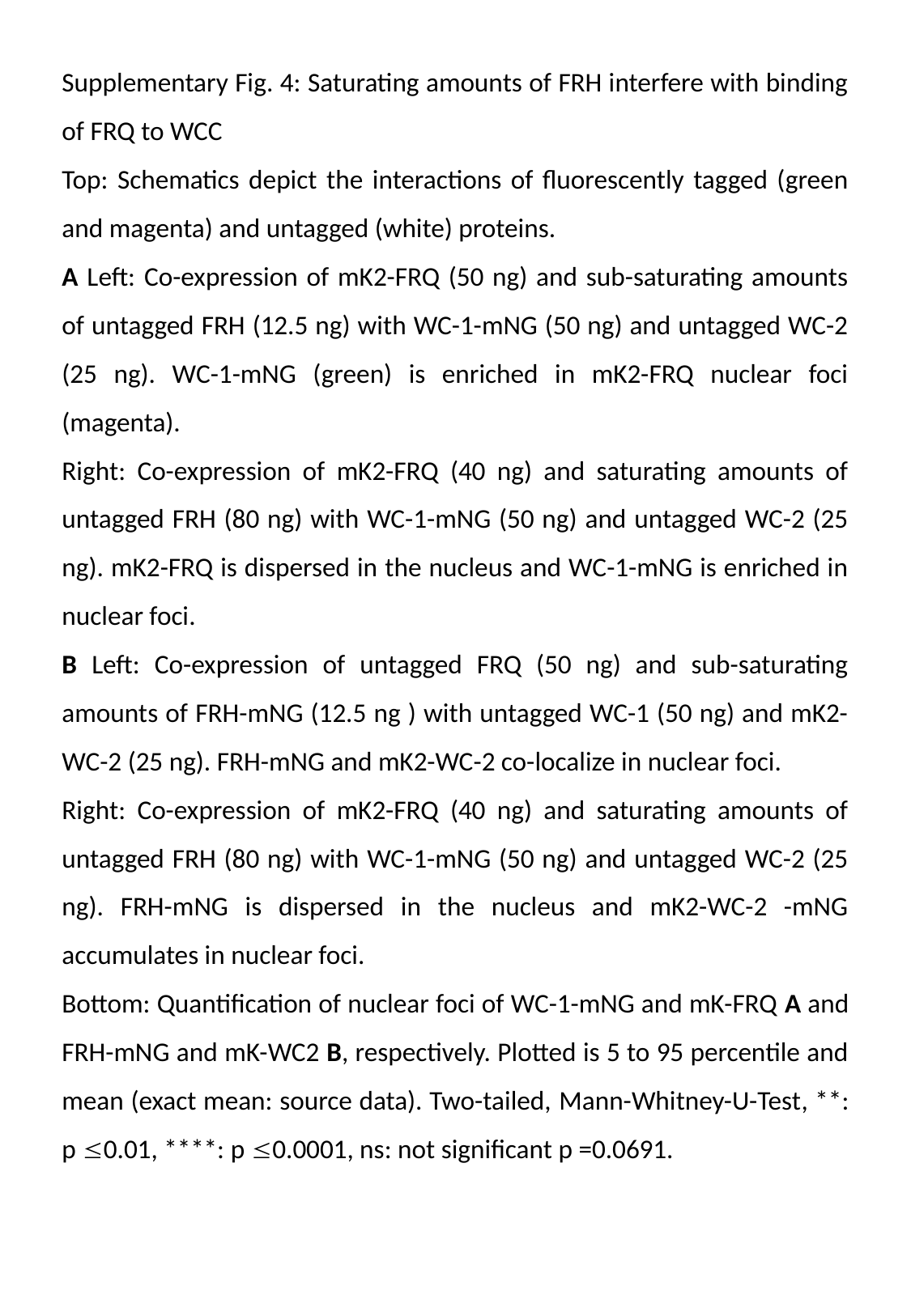

Supplementary Fig. 4: Saturating amounts of FRH interfere with binding of FRQ to WCC
Top: Schematics depict the interactions of fluorescently tagged (green and magenta) and untagged (white) proteins.
A Left: Co-expression of mK2-FRQ (50 ng) and sub-saturating amounts of untagged FRH (12.5 ng) with WC-1-mNG (50 ng) and untagged WC-2 (25 ng). WC-1-mNG (green) is enriched in mK2-FRQ nuclear foci (magenta).
Right: Co-expression of mK2-FRQ (40 ng) and saturating amounts of untagged FRH (80 ng) with WC-1-mNG (50 ng) and untagged WC-2 (25 ng). mK2-FRQ is dispersed in the nucleus and WC-1-mNG is enriched in nuclear foci.
B Left: Co-expression of untagged FRQ (50 ng) and sub-saturating amounts of FRH-mNG (12.5 ng ) with untagged WC-1 (50 ng) and mK2-WC-2 (25 ng). FRH-mNG and mK2-WC-2 co-localize in nuclear foci.
Right: Co-expression of mK2-FRQ (40 ng) and saturating amounts of untagged FRH (80 ng) with WC-1-mNG (50 ng) and untagged WC-2 (25 ng). FRH-mNG is dispersed in the nucleus and mK2-WC-2 -mNG accumulates in nuclear foci.
Bottom: Quantification of nuclear foci of WC-1-mNG and mK-FRQ A and FRH-mNG and mK-WC2 B, respectively. Plotted is 5 to 95 percentile and mean (exact mean: source data). Two-tailed, Mann-Whitney-U-Test, **: p 0.01, ****: p ≤0.0001, ns: not significant p =0.0691.

## Slide 11
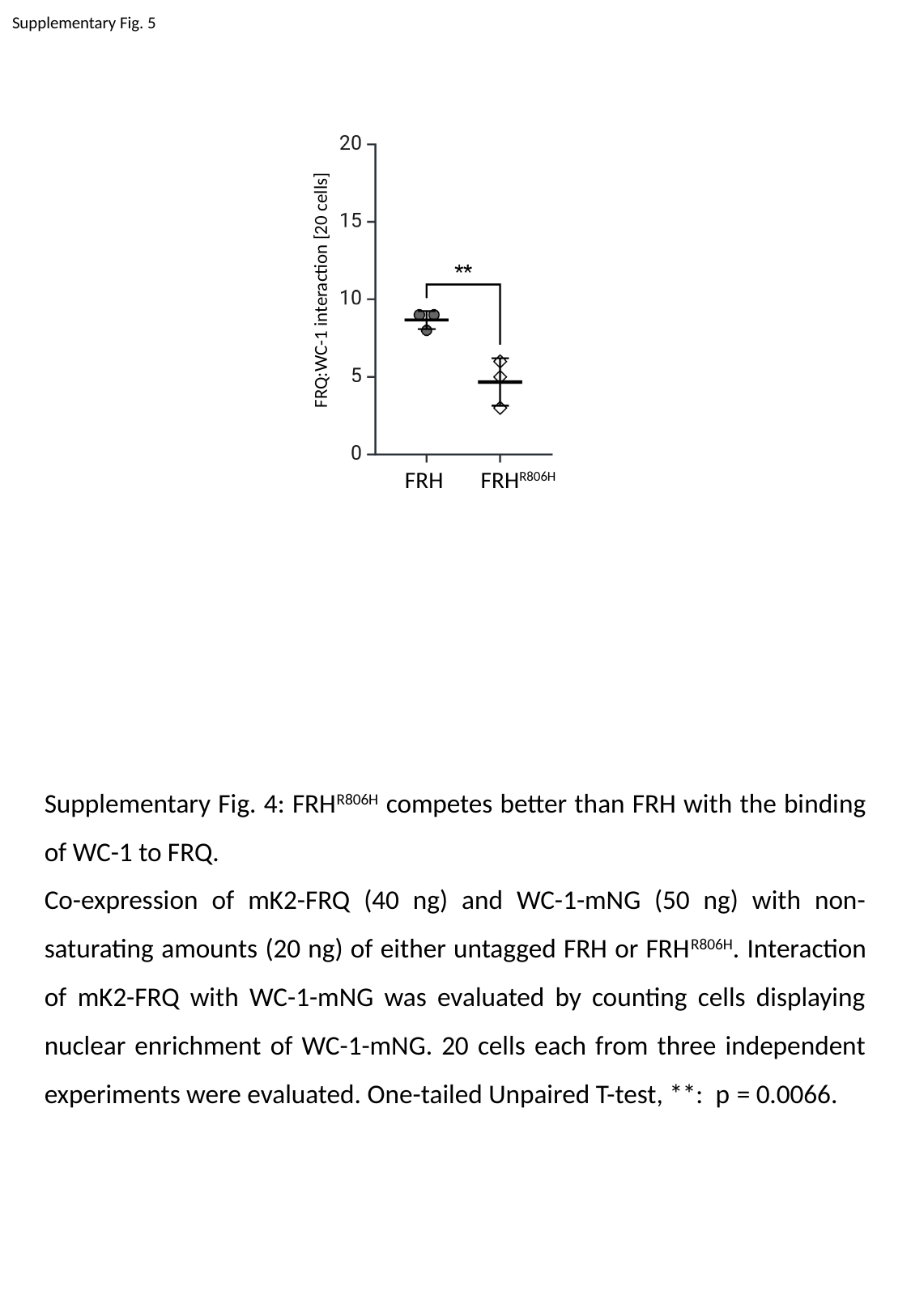

Supplementary Fig. 5
FRHR806H
FRH
FRQ:WC-1 interaction [20 cells]
Supplementary Fig. 4: FRHR806H competes better than FRH with the binding of WC-1 to FRQ.
Co-expression of mK2-FRQ (40 ng) and WC-1-mNG (50 ng) with non-saturating amounts (20 ng) of either untagged FRH or FRHR806H. Interaction of mK2-FRQ with WC-1-mNG was evaluated by counting cells displaying nuclear enrichment of WC-1-mNG. 20 cells each from three independent experiments were evaluated. One-tailed Unpaired T-test, **: p = 0.0066.

## Slide 12
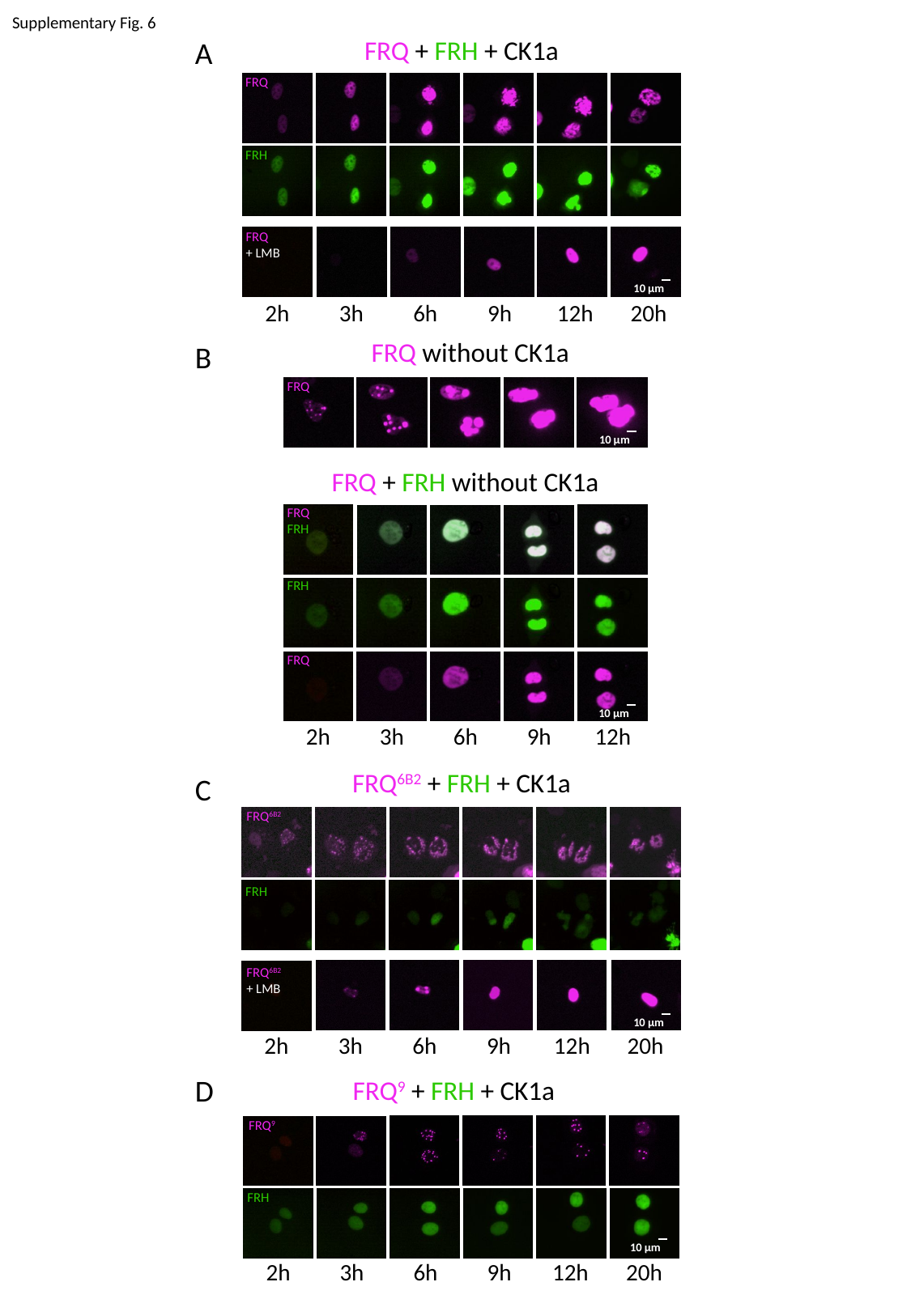

Supplementary Fig. 6
FRQ + FRH + CK1a
FRQ
FRH
FRQ
+ LMB
10 µm
2h
3h
6h
9h
12h
20h
A
FRQ without CK1a
FRQ
10 µm
10 µm
FRQ + FRH without CK1a
FRQ
FRH
FRH
FRQ
10 µm
2h
3h
6h
9h
12h
B
FRQ6B2 + FRH + CK1a
FRQ6B2
FRH
FRQ6B2
+ LMB
10 µm
2h
3h
6h
9h
12h
20h
FRQ9 + FRH + CK1a
FRQ9
FRH
10 µm
2h
3h
6h
9h
12h
20h
C
D

## Slide 13
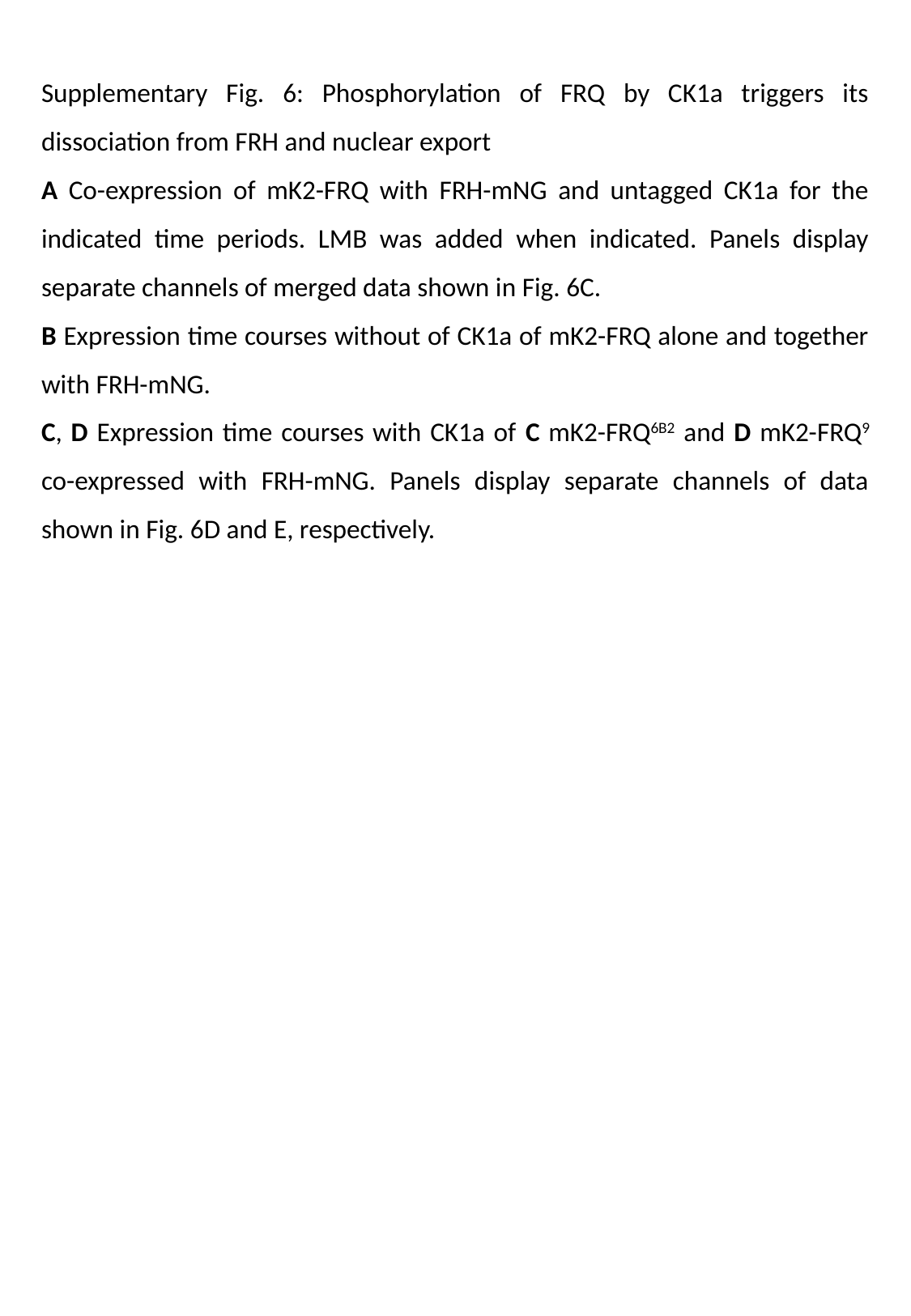

Supplementary Fig. 6: Phosphorylation of FRQ by CK1a triggers its dissociation from FRH and nuclear export
A Co-expression of mK2-FRQ with FRH-mNG and untagged CK1a for the indicated time periods. LMB was added when indicated. Panels display separate channels of merged data shown in Fig. 6C.
B Expression time courses without of CK1a of mK2-FRQ alone and together with FRH-mNG.
C, D Expression time courses with CK1a of C mK2-FRQ6B2 and D mK2-FRQ9 co-expressed with FRH-mNG. Panels display separate channels of data shown in Fig. 6D and E, respectively.

## Slide 14
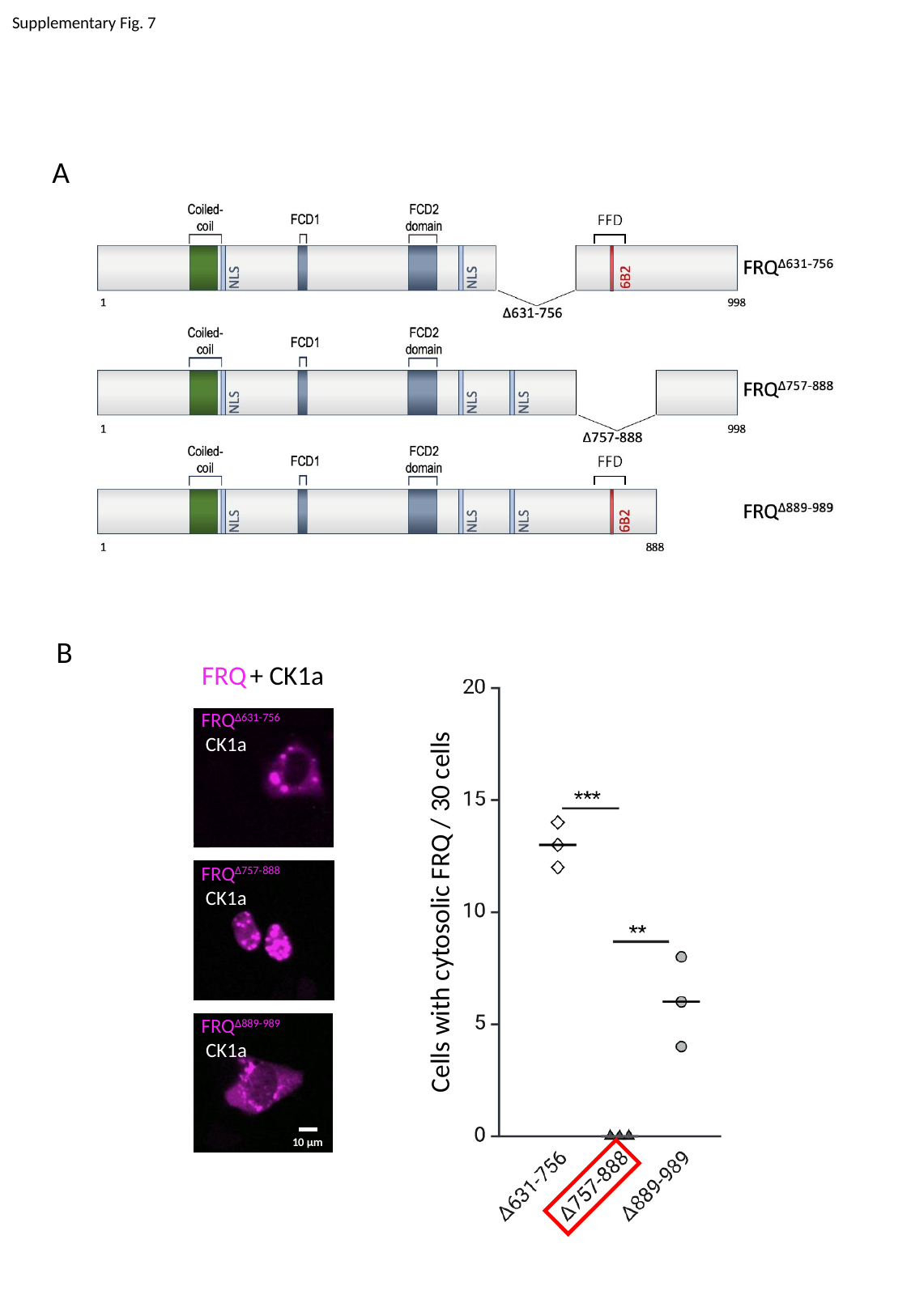

Supplementary Fig. 7
A
B
FRQ + CK1a
FRQΔ631-756
 CK1a
FRQΔ757-888
 CK1a
Cells with cytosolic FRQ / 30 cells
FRQΔ889-989
 CK1a
10 µm

## Slide 15
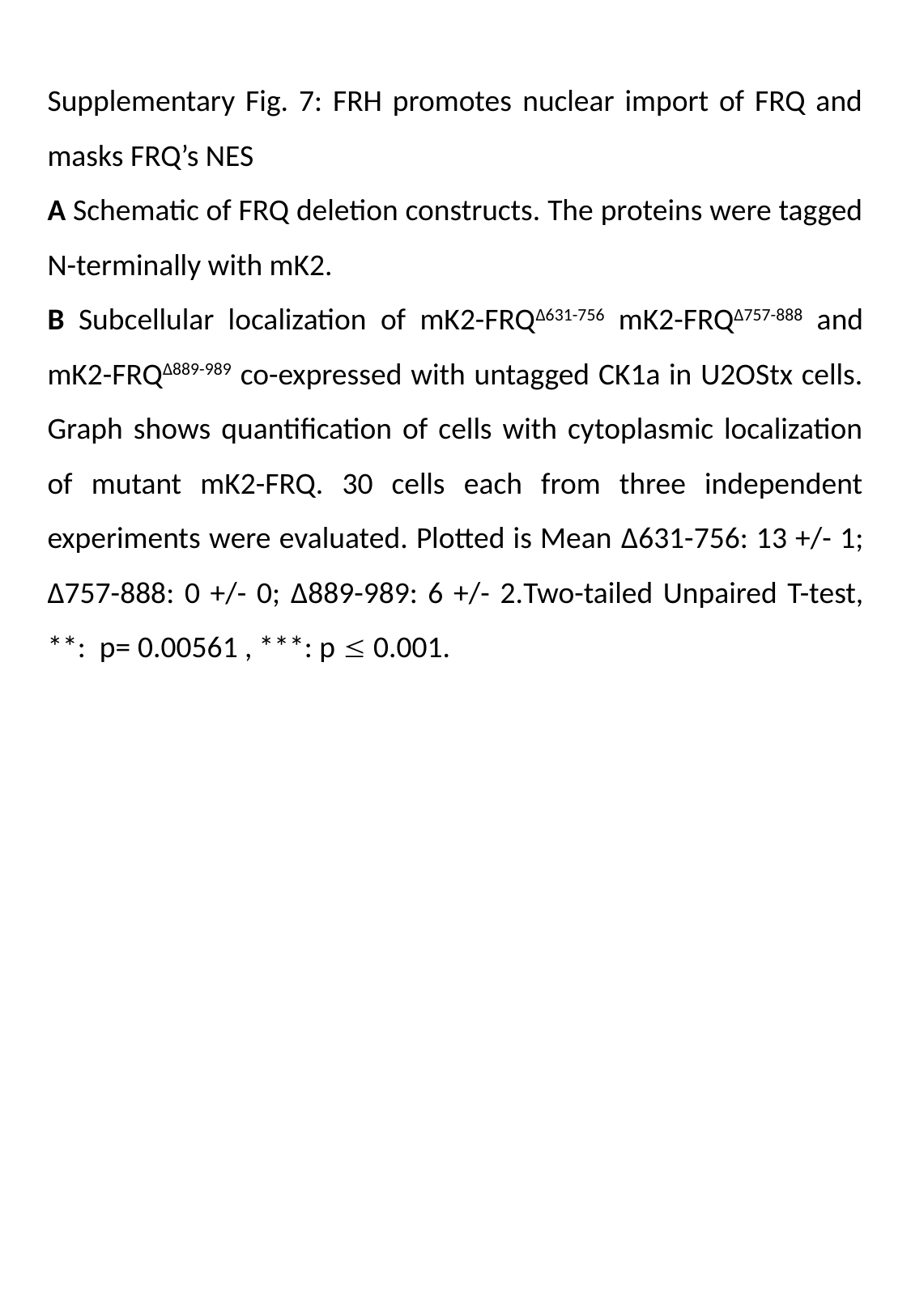

Supplementary Fig. 7: FRH promotes nuclear import of FRQ and masks FRQ’s NES
A Schematic of FRQ deletion constructs. The proteins were tagged N-terminally with mK2.
B Subcellular localization of mK2-FRQΔ631-756 mK2-FRQΔ757-888 and mK2-FRQΔ889-989 co-expressed with untagged CK1a in U2OStx cells. Graph shows quantification of cells with cytoplasmic localization of mutant mK2-FRQ. 30 cells each from three independent experiments were evaluated. Plotted is Mean Δ631-756: 13 +/- 1; Δ757-888: 0 +/- 0; Δ889-989: 6 +/- 2.Two-tailed Unpaired T-test, **: p= 0.00561 , ***: p ≤ 0.001.

## Slide 16
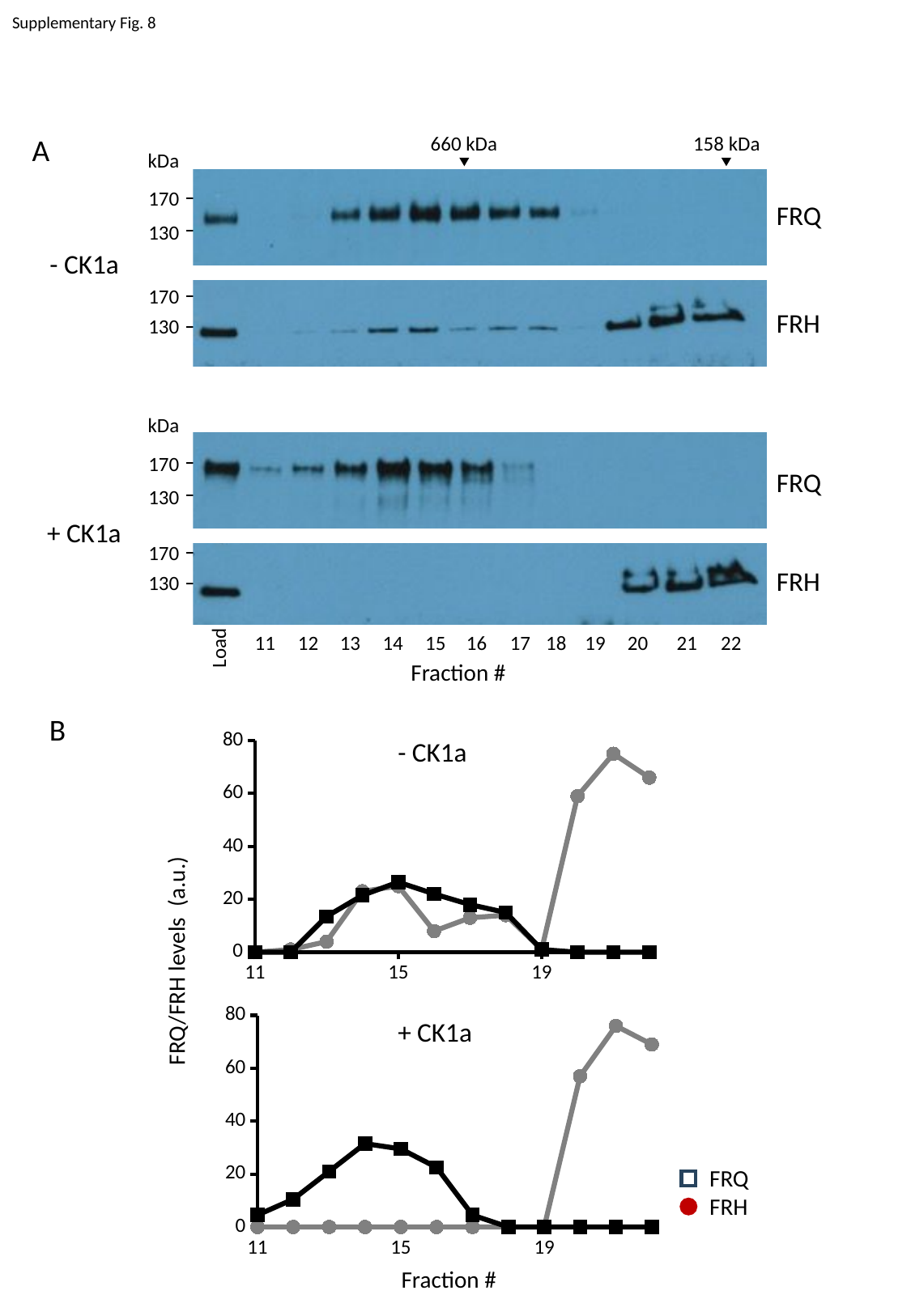

Supplementary Fig. 8
A
158 kDa
660 kDa
kDa
170
130
170
130
kDa
170
130
170
130
FRQ
- CK1a
FRH
FRQ
+ CK1a
FRH
11
12
13
14
15
16
17
18
19
20
21
22
Load
Fraction #
B
### Chart
| Category | FRQ -CK1a | FRH -CK1a |
|---|---|---|- CK1a
FRQ/FRH levels (a.u.)
### Chart
| Category | FRQ +CK1a | FRH +CK1a |
|---|---|---|+ CK1a
FRQ
FRH
Fraction #

## Slide 17
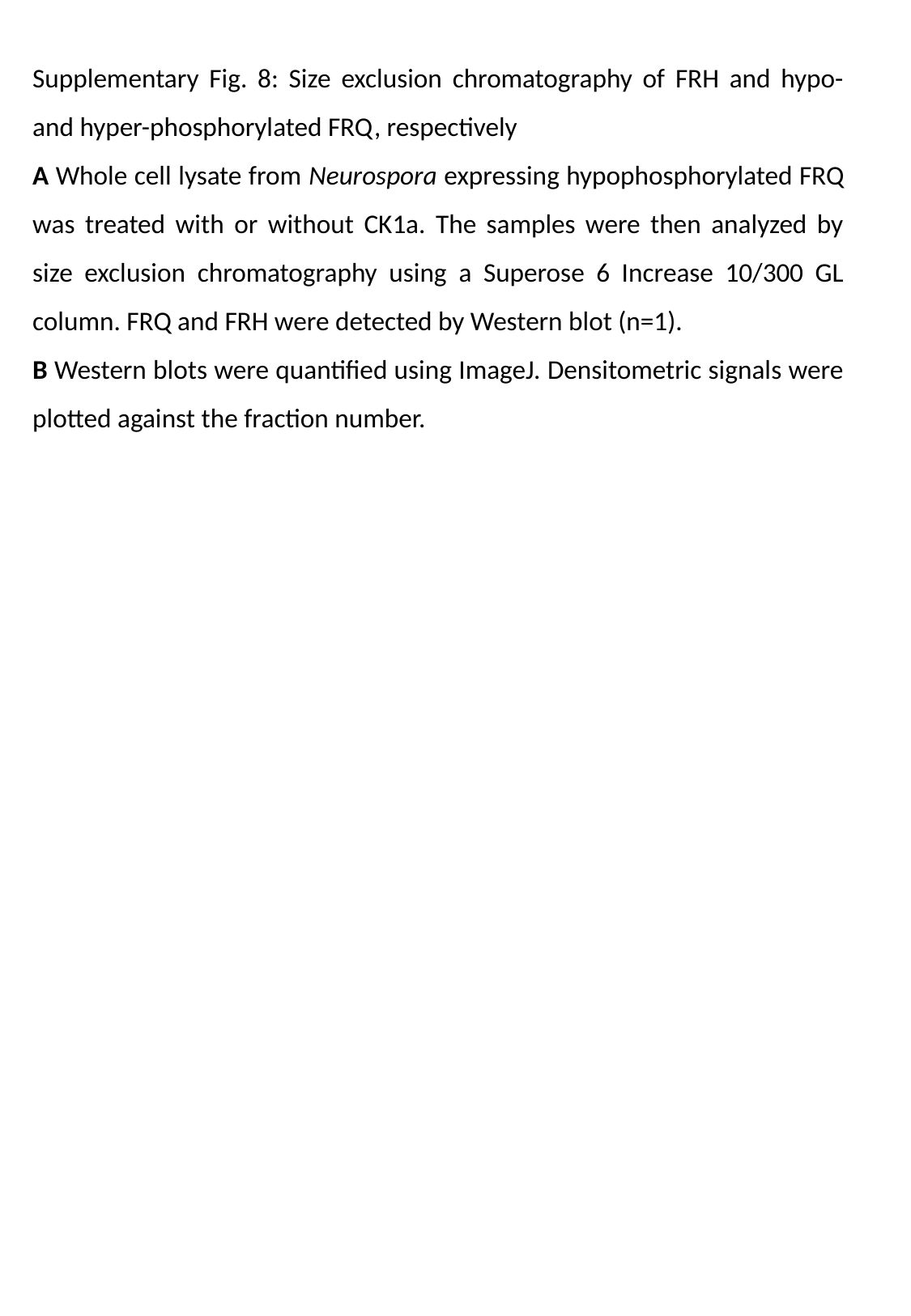

Supplementary Fig. 8: Size exclusion chromatography of FRH and hypo- and hyper-phosphorylated FRQ, respectively
A Whole cell lysate from Neurospora expressing hypophosphorylated FRQ was treated with or without CK1a. The samples were then analyzed by size exclusion chromatography using a Superose 6 Increase 10/300 GL column. FRQ and FRH were detected by Western blot (n=1).
B Western blots were quantified using ImageJ. Densitometric signals were plotted against the fraction number.
