# Supplementary material for: Casein kinase 1a mediates a two-step subunit remodeling mechanism to regulate the FRQ-FRH circadian clock complex
Source: Nat Commun. 2026 Jan 8;17:418. doi: 10.1038/s41467-025-68087-4 (PMC12796302; doi:10.1038/s41467-025-68087-4)
Supplement: Supplementary file 3 — Description of Additional Supplementary File [file 41467_2025_68087_MOESM3_ESM.pdf]

Description of Additional Supplementary Files

Supplementary Data 1:

Oligonucleotides used in this study
